# Efficacy of Non-invasive Biomarkers in Diagnosing Non-alcoholic Fatty Liver Disease (NAFLD) and Predicting Disease Progression: A Systematic Review

**DOI:** 10.7759/cureus.78421

**Published:** 2025-02-03

**Authors:** Sheenam Garg, Mansey Varghese, Fahmida Shaik, FNU Jatin, Dheerja Sachdeva, Fathima Wafa Eranhikkal, Sweta Sahu, Salma Younas

**Affiliations:** 1 Medicine, Punjab Institute of Medical Sciences, Jalandhar, IND; 2 Medicine, SMBT Institute of Medical Sciences and Research Centre, Nashik, IND; 3 Medical Education, Gandhi Medical College, Secunderabad, IND; 4 Internal Medicine, Maharaja Agrasen Medical College, Sonipat, IND; 5 Internal Medicine, Hamdard Institute of Medical Sciences and Research, New Delhi, IND; 6 Medicine, Teaching University Geomedi, Tablisi, GEO; 7 Internal Medicine, J.J.M. Medical College, Davanagere, IND; 8 Pharmacy, Punjab University College of Pharmacy, Lahore, PAK

**Keywords:** diagnosis, diagnostic efficacy, efficacy, non-alcoholic fatty liver disease (nafld), non-invasive biomarker

## Abstract

Non-alcoholic fatty liver disease (NAFLD) is a leading cause of chronic liver disease, with significant global prevalence and a strong association with metabolic syndrome, obesity, and diabetes. Early diagnosis and prediction of disease progression are critical for effective management. Non-invasive biomarkers have emerged as promising alternatives to liver biopsy, offering safer and more accessible diagnostic and prognostic options. This systematic review evaluates the efficacy of non-invasive biomarkers in diagnosing NAFLD and predicting disease progression, focusing on diagnostic accuracy, clinical utility, and limitations. A systematic review was conducted following Preferred Reporting Items for Systematic Reviews and Meta-Analyses (PRISMA) guidelines, including studies published between 2010 and 2024. Databases such as PubMed, Embase, and Scopus were searched using relevant keywords and Boolean operators. Inclusion criteria comprised studies evaluating adults (18+) with NAFLD using non-invasive biomarkers, compared to liver biopsy or other standards, and reporting diagnostic metrics such as sensitivity, specificity, and area under the curve (AUC). Data were extracted systematically, and study quality was assessed using QUADAS-2 (Quality Assessment of Diagnostic Accuracy Studies) and the Newcastle-Ottawa Scale. The nine studies include a range of biomarkers such as serum markers (Pro-C3, NIS4), imaging techniques (MRI-PDFF, cT1), and composite scores (cTAG, NFS). Diagnostic accuracy was high, with area under the curve (AUC) values ranging from 0.81 to 0.90 for detecting significant fibrosis and at-risk non-alcoholic steatohepatitis (NASH). Imaging tools such as MRI-PDFF offered superior reproducibility and whole-liver assessments, while composite biomarkers such as NIS4 demonstrated robust sensitivity but moderate specificity. Notable heterogeneity in populations and methodologies was observed. Non-invasive biomarkers show comparable diagnostic performance to liver biopsy while offering significant advantages in safety, scalability, and patient adherence. However, gaps remain, including the need for validation in diverse populations and improved specificity for advanced fibrosis. Integrating non-invasive biomarkers into clinical practice could revolutionize NAFLD management by enabling early diagnosis, guiding treatment, and reducing reliance on invasive methods. Future research should focus on validating these tools across diverse cohorts and developing novel biomarkers to address existing limitations.

## Introduction and background

Non-alcoholic fatty liver disease (NAFLD) is a chronic liver condition characterized by excessive fat accumulation in the liver, occurring in the absence of significant alcohol consumption or other secondary causes of hepatic steatosis. Globally, NAFLD affects an estimated 32% of the adult population, with higher prevalence rates reported in regions such as the Middle East (31.8%) and South America (35.7%) [1-3]. This rising prevalence correlates with the growing global burden of obesity and diabetes, making NAFLD one of the most common causes of chronic liver disease.

NAFLD and Public Health Impact

The public health implications of NAFLD are profound. The condition is strongly associated with metabolic syndrome, which is a cluster of conditions, including central obesity, hypertension, hyperglycemia, and dyslipidemia. Metabolic syndrome affects up to 90% of NAFLD patients [4]. NAFLD is also a significant driver of type 2 diabetes, with studies showing that individuals with NAFLD are two to five times more likely to develop diabetes than those without the condition [5]. Moreover, NAFLD patients face a 50% increased risk of cardiovascular diseases, which are the leading cause of death among this population [6].

Clinical Spectrum of NAFLD

NAFLD exists along a clinical spectrum, ranging from simple hepatic steatosis (fat accumulation in liver cells without inflammation) to non-alcoholic steatohepatitis (NASH), a progressive form characterized by liver inflammation, hepatocyte ballooning, and fibrosis [7]. Approximately 20-30% of individuals with NAFLD progress to NASH, and among those with NASH, 40% develop fibrosis. Advanced fibrosis, in turn, significantly increases the risk of cirrhosis and hepatocellular carcinoma (HCC). The cumulative 10-year risk of developing cirrhosis among patients with advanced fibrosis ranges from 10-15% [8,9].

NAFLD is the second leading cause of liver transplantation in the United States, with rates expected to rise further as obesity and diabetes become more prevalent. By 2030, the number of individuals with NAFLD-related cirrhosis is projected to increase by 168%, and those with NAFLD-related HCC by 137% compared to 2016 [1].

Epidemiological data and progression of NAFLD can guide public health interventions and prioritizing research into non-invasive diagnostic tools and therapeutic strategies to mitigate its systemic and economic burden.

The Need for Non-invasive Biomarkers in NAFLD

The liver biopsy is currently considered the gold standard for diagnosing NAFLD, particularly for distinguishing between simple steatosis, NASH, and stages of fibrosis. However, this procedure comes with significant limitations. A liver biopsy involves the extraction of a small liver tissue sample via a needle, which can cause discomfort, bleeding, or complications in about 0.8% and 1.8% of cases [10]. Liver biopsies are costly, with expenses varying widely but averaging between $1,500 and $3,000 per procedure in developed countries. This financial burden limits its accessibility, particularly in resource-limited settings [11]. A liver biopsy is prone to sampling variability, as the small tissue sample may not fully represent the extent of disease across the liver. Studies suggest that discrepancies in diagnosing NASH or fibrosis can occur in up to 25% of cases due to sampling errors [12]. These limitations highlight the need for alternative diagnostic methods that are less invasive, more cost-effective, and capable of providing reliable results across the entire spectrum of NAFLD.

Importance of Early and Accurate Diagnosis

Early and accurate diagnosis of NAFLD plays a role in halting disease progression. Patients with advanced fibrosis or NASH have significantly higher risks of liver-related morbidity and mortality compared to those with simple steatosis [13]. Early detection allows for timely lifestyle interventions, such as weight loss and dietary changes, which have been shown to improve liver histology and reduce fibrosis progression [14].

Moreover, biomarkers play a role in monitoring disease progression and assessing therapeutic responses. Current imaging modalities such as transient elastography and MRI, combined with blood-based biomarkers, offer promising approaches for tracking liver fibrosis and inflammation without requiring repeat biopsies [15].

Potential of Non-invasive Biomarkers

Non-invasive biomarkers hold immense potential to address the limitations of a liver biopsy and improve patient outcomes. Biomarkers such as serum tests (e.g., Pro-C3, NIS4) and imaging tools (e.g., MRI-PDFF, elastography) are easier to administer and more scalable, enabling widespread screening and monitoring of NAFLD in both clinical and community settings [16]. Advances in multi-parametric tools combining imaging and serum biomarkers have demonstrated diagnostic accuracy comparable to biopsy for detecting NASH and fibrosis. For instance, the composite cTAG score combining cT1, AST, and fasting glucose achieves an AUC of 0.90 for identifying patients with advanced fibrosis [17]. Non-invasive biomarkers are less expensive than biopsies and reduce the need for repeated procedures, lowering the overall financial burden on healthcare systems.

**Figure 1:**
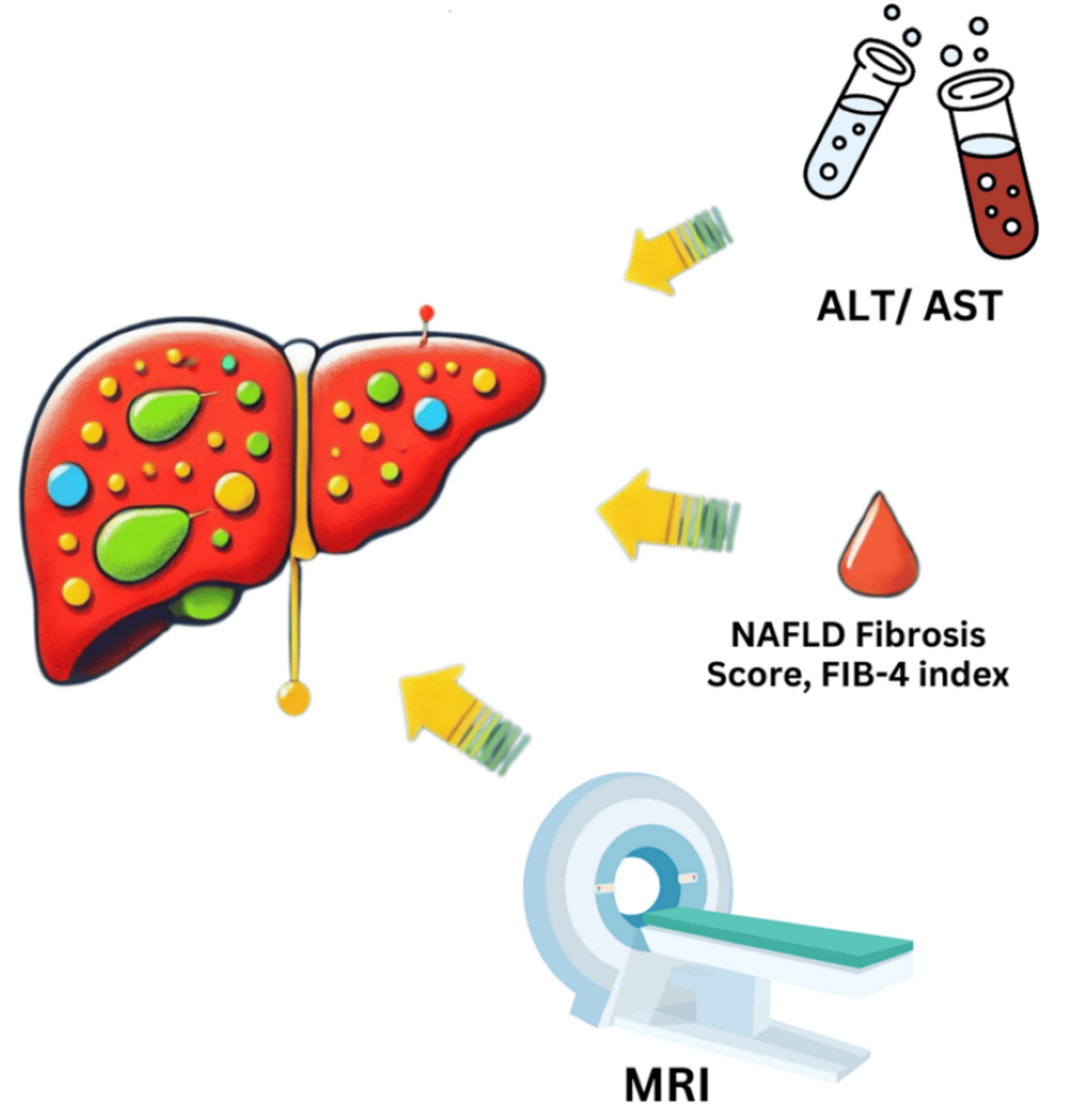
Some non-invasive biomarkers for NAFLD NAFLD: Non-alcoholic fatty liver disease Image credits: Sheenam Garg, Sweta Sahu, and Salma Younas

Non-invasive biomarkers for NAFLD encompass serum-based indicators (e.g., alanine alanine aminotransaminase (ALT), serum aspartate aminotransferase (AST), cytokeratin-18), imaging modalities (e.g., transient elastography, MRI), and clinical prediction models (e.g., NAFLD fibrosis score, FIB-4 index) (Figure 1).

Impact on Healthcare

The integration of non-invasive biomarkers into clinical practice has the potential to transform NAFLD management by enabling early diagnosis, improving patient stratification for treatments, and reducing unnecessary biopsies. These advancements can alleviate the growing economic and healthcare burden posed by NAFLD, particularly in regions with limited resources. By addressing the gaps in current diagnostic practices, non-invasive biomarkers pave the way for more effective and patient-friendly approaches to managing NAFLD and its complications [18,19].

The purpose of this review is to evaluate the efficacy of non-invasive biomarkers in the diagnosis and prediction of disease progression in NAFLD. As NAFLD becomes increasingly prevalent globally, identifying accurate and non-invasive diagnostic tools is critical for early intervention and effective disease management.

## Review

Methodology

This systematic review adhered to the Preferred Reporting Items for Systematic Reviews and Meta-Analyses (PRISMA) guidelines to ensure rigor and transparency.

Eligibility Criteria

Studies involving adults aged 18 years and older with suspected or diagnosed non-alcoholic fatty liver disease (NAFLD) were included. The studies assessed the use of non-invasive biomarkers, including serum markers, imaging techniques, and scoring systems, for diagnosing NAFLD and predicting disease progression. Comparators included liver biopsy, other biomarkers, or no comparator. Key outcomes evaluated were diagnostic accuracy metrics, such as sensitivity, specificity, and area under the curve (AUC), as well as outcomes related to disease progression, including fibrosis stages and cirrhosis. Eligible studies included observational studies, diagnostic accuracy studies, clinical trials, and systematic reviews or meta-analyses published in English between 2010 and 2024.

Exclusion Criteria

Studies focusing on pediatric populations, other liver diseases (e.g., alcoholic liver disease, viral hepatitis), and non-peer-reviewed studies or conference abstracts were excluded.

Search Strategy

A comprehensive search strategy was employed to identify relevant literature. The databases searched included PubMed, Embase, Web of Science, Scopus, and the Cochrane Library. Keywords and MeSH terms were selected to maximize the retrieval of pertinent studies, using terms such as “non-invasive biomarkers”, “non-alcoholic fatty liver disease”, “NAFLD diagnosis”, “liver fibrosis biomarkers”, and “predictive biomarkers for NAFLD”. Boolean operators, including “OR” and “AND”, were applied to refine the search results. To ensure the inclusion of all relevant articles, the reference lists of included studies were manually screened for additional citations.

Study Selection and Screening Process

The study selection process involved two distinct phases. Initially, two independent reviewers conducted title and abstract screening to identify studies that met the eligibility criteria. In the second phase, the full texts of studies deemed relevant were reviewed to confirm their inclusion. In cases of disagreement during the selection process, a third reviewer was consulted to resolve disputes and ensure consistency in decision-making.

**Figure 2:**
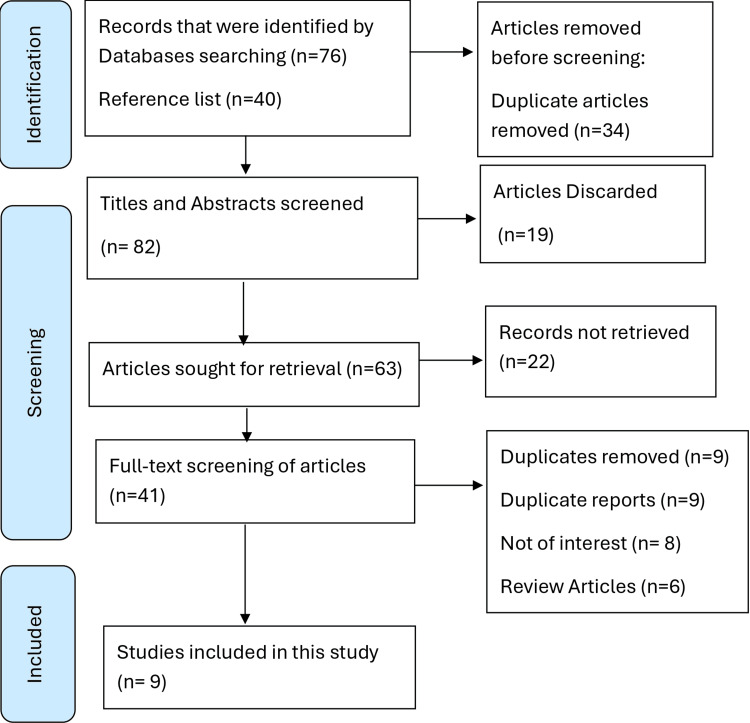
PRISMA flow diagram PRISMA: Preferred Reporting Items for Systematic Reviews and Meta-Analyses

The PRISMA flow diagram represents the whole process of study selection and screening (Figure 2).

Data Extraction

Variables extracted included study details (e.g., authors, publication year, design, sample size), population characteristics (e.g., age, sex, disease severity), and biomarkers assessed (e.g., serum markers such as ALT, AST, FibroScan, NFS, and imaging modalities such as MRI-PDFF and elastography). Diagnostic accuracy metrics, including sensitivity, specificity, and AUC, were also recorded. In addition, outcomes related to disease progression, such as fibrosis stages and the development of cirrhosis, were documented. The extraction process also accounted for methodological limitations or biases identified in each study (Table 1).

**Table 1:** Characteristics of the studies AUC: Area Under the Curve; NAFLD: Non-alcoholic Fatty Liver Disease; NASH: Non-alcoholic Steatohepatitis; CK-18-M30: Cytokeratin-18 (M30 fragment); FGF-21: Fibroblast Growth Factor 21; IL-1Ra: Interleukin-1 Receptor Antagonist; PEDF: Pigment Epithelium-Derived Factor; OPG: Osteoprotegerin; cT1: Corrected T1 (T1-Weighted MRI Parameter); NAS: NAFLD Activity Score; MRI-PDFF: Magnetic Resonance Imaging-Proton Density Fat Fraction; Pro-C3: Pro-Collagen III N-Terminal Peptide; NIS4: Non-invasive Scoring 4; HbA1c: Hemoglobin A1c; AST: Aspartate Aminotransferase; cTAG: Corrected TAG (T1-Weighted MRI Parameter Combined with AST and Glucose); FIBC3: Fibrosis-Corrected Biomarker Combination 3; ABC3D: Advanced Biomarker Combination for Disease Diagnosis; ADAMTSL2: A Disintegrin and Metalloproteinase with Thrombospondin Motifs-Like Protein 2

Study	Type	Sample Size	Population Characteristics	Biomarkers Assessed	Diagnostic Accuracy Metrics (Sensitivity, Specificity, AUC)	Outcomes Related to Disease Progression	Limitations and Biases
Yang et al., 2015 [20]	Observational study	179 patients, 91 controls, 63 validation group	Adults with biopsy-proven NAFLD; controls age- and sex-matched	Cytokeratin-18 (CK-18-M30), FGF-21, IL-1Ra, PEDF, OPG	AUCs ranged from 0.86 to 0.89; CK-18-M30 sensitivity 70%, specificity 79%	Improved accuracy in diagnosing NASH when combining biomarkers	Small validation group; limited generalizability
Andersson et al., 2021 [21]	Pooled analysis and meta-analysis	543 participants	Suspected NAFLD; focus on NASH and fibrosis (NAS ≥4, F ≥2)	MRI-derived cT1, liver fat content	cT1: AUC 0.78 (95% CI: 0.74-0.82); Combined cT1 and liver fat AUC 0.82 (95% CI: 0.78-0.85)	Identified 'high-risk' NASH and fibrosis with cT1	Pooled data lacked external validation for combined biomarkers
Mak et al., 2021 [16]	Systematic review/meta-analysis	1568-2058 patients across studies	Adults with NAFLD, no coexisting liver diseases	Pro-C3	Pro-C3: Significant fibrosis AUC 0.81 (95% CI: 0.77-0.84), advanced fibrosis AUC 0.79 (95% CI: 0.73-0.82)	Pro-C3 supports non-invasive fibrosis staging	High heterogeneity among included studies
Harrison et al., 2020 [22]	Prospective validation	702 patients across three cohorts	Suspected NAFLD; metabolic risk factors	NIS4 panel (miR-34a-5p, alpha-2 macroglobulin, YKL-40, HbA1c)	NIS4: Sensitivity 81.5% (95% CI: 76.9-85.3%), specificity 63.0% (95% CI: 57.8-68.0%), AUC 0.83 (95% CI: 0.79-0.86)	Effective non-invasive identification of at-risk NASH (NAS â‰¥4, F â‰¥2)	Moderate specificity could result in missed at-risk NASH cases
Ajmera et al., 2021 [15]	Review	Not applicable	Broad NAFLD population with diverse disease stages	MRI-PDFF, elastography	Elastography: AUC ~0.9; MRI-PDFF superior for detecting longitudinal changes in liver fat	Demonstrated longitudinal utility of MRI biomarkers	Standalone imaging biomarkers under investigation; modest diagnostic accuracy for NASH
Dennis et al., 2020 [17]	Retrospective cohort	86 biopsy-confirmed NAFLD patients	Adults, varying fibrosis stages	cT1, AST, fasting glucose (cTAG score)	cTAG: AUC 0.90 (95% CI: 0.84-0.97)	Improved screening for trial-eligible NASH patients	Retrospective design; small sample size
Boyle et al., 2019 [23]	Validation study	449 patients	Biopsy-confirmed NAFLD, all disease stages	PRO-C3, FIBC3, ABC3D scores	FIBC3: AUC 0.89 (discovery), 0.83 (validation); ABC3D: AUC 0.88 (discovery), 0.81 (validation)	Improved advanced fibrosis detection using validated scores	Complex scoring may hinder routine clinical use
Corey et al., 2021 [24]	Aptamer-based proteomics	398 patients across cohorts	Adults with varying fibrosis stages	ADAMTSL2, 8-protein panel	ADAMTSL2: AUC 0.83-0.86; 8-protein panel: AUC 0.90 (Cohorts C and D)	Highly accurate identification of at-risk NASH and fibrosis	Limited demographic diversity in study cohorts
Perakakis et al., 2019 [25]	Proof-of-concept study	80 participants	Biopsy-confirmed NAFLD; 49 healthy, 31 with NAFL or NASH	Lipids, glycans, hormones	Fibrosis detection: AUC 0.98; accuracy up to 90% for differentiating NAFL from NASH	Lipidomic signatures enable accurate fibrosis diagnosis	Small sample size; external validation needed

Quality Assessment

We used QUADAS-2 (Quality Assessment of Diagnostic Accuracy Studies) tool to assess diagnostic accuracy studies included in this review. Results were summarized in a tabular format indicating the level of bias (low, high, unclear) in each domain for each diagnostic study.

The Newcastle-Ottawa Scale (NOS) was employed to evaluate the quality of observational studies. Each study was assigned a score out of nine, with higher scores reflecting better methodological quality. Studies scoring 7-9 were categorized as high quality, 5-6 as moderate quality, and <5 as low quality.

The table details the risk of bias for various domains and provides an overall quality assessment for each study (Table 2).

**Table 2:** Risk of bias and quality assessment QUADAS-2: Quality Assessment of Diagnostic Accuracy Studies

Study	Tool Used	Risk of Bias (Patient	Risk of Bias (Index Test/Comparability)	Risk of Bias (Reference Standard/Outcome Assessment)	Risk of Bias (Flow and Timing)	NOS Score (Observational Studies)	Overall Quality Assessment
Yang et al., 2015 [20]	QUADAS-2	Low	Moderate (blinding unclear)	High (variability in biopsy interpretation)	Moderate (missing follow-up data)	Not Applicable	Moderate
Andersson et al., 2021 [21]	QUADAS-2	Low	Low	Moderate	Low	Not Applicable	High
Mak et al., 2021 [16]	Newcastle-Ottawa Scale	High (non-representative sample)	Moderate (limited confounder adjustment)	Low	Low	6/9	Moderate
Harrison et al., 2020 [22]	QUADAS-2	Low	Low	Moderate (liver biopsy standard questioned)	Moderate	Not Applicable	Moderate
Ajmera et al., 2021 [15]	Review (Not Scored)	Not Applicable	Not Applicable	Not Applicable	Not Applicable	Not Applicable	Not Scored
Dennis et al., 2020 [17]	QUADAS-2	Low	Moderate (blinding unclear)	Moderate	Moderate	Not Applicable	Moderate
Boyle et al., 2019 [23]	QUADAS-2	Moderate	Low	Moderate	Moderate	Not Applicable	Moderate
Corey et al., 2021 [24]	QUADAS-2	Low	Low	Low	Low	Not Applicable	High
Perakakis et al., 2019 [25]	QUADAS-2	Moderate	Moderate	Low	Low	Not Applicable	Moderate

Results

This systematic review included a total of nine studies, comprising diagnostic accuracy studies, observational studies, and a systematic review. The studies varied in their design and objectives, but all focused on the evaluation of non-invasive biomarkers for diagnosing NAFLD and predicting disease progression. Geographically, the studies were conducted across various regions, including North America, Europe, and Asia, reflecting a diverse population base. The sample sizes ranged from 80 to over 2,000 participants, with most studies involving adults with biopsy-NAFLD.

Key Findings

The included studies investigated a variety of non-invasive biomarkers, which were broadly categorized into the following groups. Frequently studied biomarkers included ALT, AST, and advanced fibrosis-related markers, such as Pro-C3, NIS4, and cytokeratin-18 (CK-18). Tools such as MRI-PDFF and FibroScan and multiparametric MRI markers such as cT1 were commonly evaluated for their diagnostic and prognostic capabilities. Clinical scoring systems, including the NAFLD fibrosis score (NFS) and FibroScan-associated elastography scores, were also assessed for their utility in staging fibrosis and predicting disease progression.

Diagnostic Performance Metrics

The diagnostic performance of the biomarkers was summarized using sensitivity, specificity, positive predictive value (PPV), negative predictive value (NPV), and area under the receiver operating characteristic curve (AUC). Below is a table summarizing these metrics for key biomarkers (Table 3).

**Table 3:** Diagnostic performance metrics AUC: Area Under the Curve; NPV: Negative Predictive Value; PPV: Positive Predictive Value; NASH: Non-alcoholic Steatohepatitis; N/A: Not Applicable

Biomarker	Sensitivity (%)	Specificity (%)	AUC	PPV	NPV	Purpose
CK-18 (Yang et al.) [20]	70	79	0.86–0.89	79	70	Diagnostic (NASH)
cT1 (Andersson et al.) [21]	76	81	0.82	N/A	N/A	Diagnostic/Prognostic
Pro-C3 (Mak et al.) [16]	80	72	0.81	N/A	N/A	Diagnostic (Fibrosis)
NIS4 (Harrison et al.) [22]	81.5	63	0.83	N/A	N/A	Diagnostic (at-risk NASH)
MRI-PDFF (Ajmera et al.) [15]	~90	~85	~0.90	N/A	N/A	Diagnostic (fat fraction)
cTAG (Dennis et al.) [17]	N/A	N/A	0.90	N/A	N/A	Diagnostic (fibrosis)

The AUC values ranged from 0.81 to 0.90, indicating good to excellent diagnostic performance for most biomarkers. Sensitivity and specificity varied based on the population and methodology, with composite scores and imaging tools generally showing higher performance metrics compared to single serum markers.

Heterogeneity in Studies

Significant heterogeneity was noted across the included studies in terms of study populations, methodologies, and reported outcomes. Differences in age, sex, and the severity of NAFLD among participants contributed to variability in the results. For example, some studies focused exclusively on biopsy-confirmed patients, while others included a broader spectrum of suspected NAFLD cases. The biomarkers evaluated and the reference standards used (e.g., liver biopsy, imaging) varied across studies. Additionally, some studies utilized advanced imaging techniques, while others relied on simpler serum-based tests. The criteria for diagnosing fibrosis, cirrhosis, or at-risk NASH were not uniform, leading to discrepancies in reported diagnostic accuracy metrics. Despite these differences, the studies collectively provide strong evidence supporting the utility of non-invasive biomarkers in diagnosing and predicting the progression of NAFLD.

Discussion

The findings of this systematic review highlight the potential of non-invasive biomarkers to transform the diagnosis and management of NAFLD. Biomarkers such as Pro-C3 and NIS4 and imaging techniques, such as MRI-PDFF and cT1, demonstrated good diagnostic accuracy with AUC values ranging from 0.81 to 0.90, placing them within the "excellent" range for diagnostic tools. For example, Pro-C3 achieved an AUC of 0.81 for detecting significant fibrosis, which is comparable to other well-established markers such as the NFS but with potentially greater sensitivity to early fibrotic changes [16].

MRI-PDFF and elastography, with AUCs approaching 0.90, represent a significant advancement in the field of non-invasive diagnostics. These tools provide consistent, reproducible data that can facilitate longitudinal tracking of disease progression. However, variability in sensitivity (e.g., NIS4 at 81.5%) and specificity (e.g., Pro-C3 at 72%) raises questions about their reliability across diverse clinical contexts. For instance, while NIS4 excels in identifying at-risk NASH, its moderate specificity (63%) suggests a higher likelihood of false positives in some populations [22].

Strengths and Limitations

The studies reviewed possess notable strengths. Large, multi-cohort analyses, such as those involving over 2,000 participants in systematic reviews, provide robust pooled estimates that improve generalizability [16]. Innovative methodologies, such as multiparametric imaging and the use of composite scores such as cTAG, represent significant advancements in diagnostic research. For instance, cTAG demonstrated an AUC of 0.90, placing it among the most reliable tools for assessing advanced fibrosis [17].

However, limitations were prevalent, particularly regarding heterogeneity in study design and populations. The use of different diagnostic thresholds and reference standards, such as liver biopsy, often introduced variability in reported outcomes. Additionally, studies with small sample sizes (e.g., Perakakis et al. [25], with 80 participants) may lack the power to detect nuanced differences, thereby limiting the robustness of their conclusions. The reliance on biopsy, despite its status as a gold standard, introduces potential biases related to sampling variability and interobserver differences, with reported discordance rates of up to 25% in fibrosis staging [12].

Clinical Implications

The implications for clinical practice are substantial. Non-invasive biomarkers offer a safer, more accessible alternative to liver biopsy, with MRI-PDFF and Pro-C3 emerging as frontrunners in diagnostic and prognostic applications. MRI-PDFF, with an AUC of 0.90, provides highly accurate assessments of liver fat content and shows potential for tracking longitudinal changes, making it valuable for monitoring treatment response [15]. Pro-C3, while slightly less specific, can help stratify patients into appropriate risk categories, enabling targeted interventions to mitigate fibrosis progression.

However, integrating these biomarkers into routine clinical workflows requires addressing challenges such as cost, availability, and the need for standardized diagnostic cut-offs. Furthermore, while tools such as NIS4 offer high sensitivity, their lower specificity necessitates confirmatory testing to avoid overtreatment.

Diagnostic Performance of Non-invasive Biomarkers vs. Liver Biopsy

Non-invasive biomarkers have demonstrated diagnostic performance comparable to a liver biopsy in specific scenarios, particularly for detecting fibrosis and steatosis. For instance, Pro-C3, a biomarker associated with fibrogenesis, achieved an AUC of 0.81 for significant fibrosis detection, comparable to biopsy-derived histological assessments but without the risks of invasiveness [16]. Similarly, MRI-PDFF, with an AUC of approximately 0.90, provides an accurate measure of liver fat content and outperforms a biopsy in terms of reproducibility and the ability to capture global liver characteristics rather than localized sampling [15].

Composite biomarkers such as the NIS4 panel also rival a biopsy in identifying at-risk NASH (NAS ≥4, F ≥2), with a sensitivity of 81.5% and specificity of 63% [22]. While biopsy remains a gold standard, its reliance on localized tissue sampling introduces variability, with discordance rates of up to 25% in fibrosis staging due to sampling error [12]. Non-invasive tools, in contrast, provide whole-liver assessments and can be used longitudinally to monitor disease progression or therapeutic responses.

Advantages of Non-invasive Biomarkers

Non-invasive biomarkers offer several advantages over liver biopsy. Biomarkers such as MRI-PDFF and Pro-C3 eliminate the risks of complications such as bleeding and infection associated with biopsy, making them safer for repeated use [26,27]. Serum-based biomarkers and imaging techniques are more scalable and can be implemented in primary care settings, addressing barriers to biopsy availability [28,29]. While initial costs for imaging tools such as MRI may be higher, the reduced need for repeat procedures and avoidance of hospitalization for biopsy-related complications make non-invasive tools more cost-effective in the long term [30].

Scenarios Where Liver Biopsy Remains Necessary

Despite the advancements in non-invasive diagnostics, liver biopsy retains its role in specific scenarios. When clinical findings and non-invasive tests yield conflicting results, a biopsy is essential to confirm the diagnosis or exclude other liver diseases. A biopsy provides granular information on inflammation, hepatocyte ballooning, and subtle fibrosis changes, which current biomarkers cannot fully replicate [31]. Biopsy remains a requirement for many clinical trials investigating NAFLD treatments, as histological endpoints are still considered the definitive measure of therapeutic efficacy [32].

Critical Limitations of Non-invasive Biomarkers

While non-invasive biomarkers rival biopsies in many aspects, they are not without limitations. Moderate specificity in some biomarkers, such as NIS4 (63%), can lead to false positives, necessitating confirmatory testing in clinical practice [22]. Variability in biomarker thresholds and diagnostic cut-offs across populations limits their universal applicability. In cases of early or subtle fibrosis, non-invasive tools may fail to detect minimal histological changes that biopsy can identify.

Identified Gaps in the Literature

Many biomarkers lack validation in diverse populations, particularly those with varying ethnicities, metabolic comorbidities, or stages of fibrosis. This limitation is problematic given the global burden of NAFLD and the need for universally applicable diagnostic tools. While biomarkers such as Pro-C3 and NIS4 excel in detecting early-stage fibrosis, fewer studies focus on predicting advanced fibrosis or cirrhosis, where the risk of liver-related mortality is the highest.

Methodological inconsistencies, such as differing definitions of fibrosis stages and variability in outcome measures, further complicate comparisons between studies. To advance the field, large-scale, multicenter trials with standardized protocols and external validation are urgently needed. Future research should also explore cost-effectiveness and the integration of biomarkers into risk prediction models for broader clinical utility.

## Conclusions

Biomarkers such as Pro-C3 and NIS4 and imaging tools such as MRI-PDFF and cT1 demonstrate robust diagnostic accuracy, providing a safe and scalable alternative to a liver biopsy. These tools offer significant advantages, including improved patient safety, greater accessibility, and enhanced reproducibility, while maintaining comparable diagnostic performance in detecting fibrosis and at-risk NASH. However, gaps remain, including the need for validation in large, multi-ethnic cohorts and the development of biomarkers with improved specificity for advanced fibrosis and cirrhosis. Future research should also focus on integrating biomarkers with imaging techniques to further enhance diagnostic accuracy and enable precise monitoring of disease progression.

## References

[1] Younossi ZM, Golabi P, Paik JM, Henry A, Van Dongen C, Henry L (2023). The global epidemiology of nonalcoholic fatty liver disease (NAFLD) and nonalcoholic steatohepatitis (NASH): a systematic review. Hepatology.

[2] Le MH, Yeo YH, Li X (2022). 2019 global NAFLD prevalence: a systematic review and meta-analysis. Clin Gastroenterol Hepatol.

[3] Quek J, Chan KE, Wong ZY (2023). Global prevalence of non-alcoholic fatty liver disease and non-alcoholic steatohepatitis in the overweight and obese population: a systematic review and meta-analysis. Lancet Gastroenterol Hepatol.

[4] Musso G, Gambino R, Cassader M, Pagano G (2011). Meta-analysis: natural history of non-alcoholic fatty liver disease (NAFLD) and diagnostic accuracy of non-invasive tests for liver disease severity. Ann Med.

[5] Nogueira JP, Cusi K (2024). Role of insulin resistance in the development of nonalcoholic fatty liver disease in people with type 2 diabetes: from bench to patient care. Diabetes Spectr.

[6] Targher G, Byrne CD, Tilg H (2020). NAFLD and increased risk of cardiovascular disease: clinical associations, pathophysiological mechanisms and pharmacological implications. Gut.

[7] Pouwels S, Sakran N, Graham Y (2022). Non-alcoholic fatty liver disease (NAFLD): a review of pathophysiology, clinical management and effects of weight loss. BMC Endocr Disord.

[8] Anstee QM, Berentzen TL, Nitze LM (2024). Prognostic utility of fibrosis-4 index for risk of subsequent liver and cardiovascular events, and all-cause mortality in individuals with obesity and/or type 2 diabetes: a longitudinal cohort study. Lancet Reg Health Eur.

[9] Innes H, Morling JR, Buch S, Hamill V, Stickel F, Guha IN (2022). Performance of routine risk scores for predicting cirrhosis-related morbidity in the community. J Hepatol.

[10] Takyar V, Etzion O, Heller T (2017). Complications of percutaneous liver biopsy with Klatskin needles: a 36-year single-centre experience. Aliment Pharmacol Ther.

[11] Allen AM, Van Houten HK, Sangaralingham LR, Talwalkar JA, McCoy RG (2018). Healthcare cost and utilization in nonalcoholic fatty liver disease: real-world data from a large U.S. claims database. Hepatology.

[12] Ratziu V, Charlotte F, Heurtier A (2005). Sampling variability of liver biopsy in nonalcoholic fatty liver disease. Gastroenterology.

[13] Alkhouri N, McCullough AJ (2012). Noninvasive diagnosis of NASH and liver fibrosis within the spectrum of NAFLD. Gastroenterol Hepatol (N Y).

[14] Makri E, Goulas A, Polyzos SA (2021). Epidemiology, pathogenesis, diagnosis and emerging treatment of nonalcoholic fatty liver disease. Arch Med Res.

[15] Ajmera V, Loomba R (2021). Imaging biomarkers of NAFLD, NASH, and fibrosis. Mol Metab.

[16] Mak AL, Lee J, van Dijk AM (2021). Systematic review with meta-analysis: diagnostic accuracy of Pro-C3 for hepatic fibrosis in patients with non-alcoholic fatty liver disease. Biomedicines.

[17] Dennis A, Mouchti S, Kelly M, Fallowfield JA, Hirschfield G, Pavlides M, Banerjee R (2020). A composite biomarker using multiparametric magnetic resonance imaging and blood analytes accurately identifies patients with non-alcoholic steatohepatitis and significant fibrosis. Sci Rep.

[18] Hernandez Roman J, Siddiqui MS (2020). The role of noninvasive biomarkers in diagnosis and risk stratification in nonalcoholic fatty liver disease. Endocrinol Diabetes Metab.

[19] Long MT, Gandhi S, Loomba R (2020). Advances in non-invasive biomarkers for the diagnosis and monitoring of non-alcoholic fatty liver disease. Metabolism.

[20] Yang M, Xu D, Liu Y (2015). Combined serum biomarkers in non-invasive diagnosis of non-alcoholic steatohepatitis. PLoS One.

[21] Andersson A, Kelly M, Imajo K (2022). Clinical utility of magnetic resonance imaging biomarkers for identifying nonalcoholic steatohepatitis patients at high risk of progression: a multicenter pooled data and meta-analysis. Clin Gastroenterol Hepatol.

[22] Harrison SA, Ratziu V, Boursier J (2020). A blood-based biomarker panel (NIS4) for non-invasive diagnosis of non-alcoholic steatohepatitis and liver fibrosis: a prospective derivation and global validation study. Lancet Gastroenterol Hepatol.

[23] Boyle M, Tiniakos D, Schattenberg JM (2019). Performance of the PRO-C3 collagen neo-epitope biomarker in non-alcoholic fatty liver disease. JHEP Rep.

[24] Corey KE, Pitts R, Lai M (2022). ADAMTSL2 protein and a soluble biomarker signature identify at-risk non-alcoholic steatohepatitis and fibrosis in adults with NAFLD. J Hepatol.

[25] Perakakis N, Polyzos SA, Yazdani A, Sala-Vila A, Kountouras J, Anastasilakis AD, Mantzoros CS (2019). Non-invasive diagnosis of non-alcoholic steatohepatitis and fibrosis with the use of omics and supervised learning: a proof of concept study. Metabolism.

[26] Abdelhameed F, Kite C, Lagojda L (2024). Non-invasive scores and serum biomarkers for fatty liver in the era of metabolic dysfunction-associated steatotic liver disease (MASLD): a comprehensive review from NAFLD to MAFLD and MASLD. Curr Obes Rep.

[27] Schaapman J, Shumbayawonda E, Castelo-Branco M (2024). MRI-serum-based score accurately identifies patients undergoing liver transplant without rejection avoiding the need for liver biopsy: a multisite European study. Liver Transpl.

[28] Stine JG, Munaganuru N, Barnard A (2021). Change in MRI-PDFF and histologic response in patients with nonalcoholic steatohepatitis: a systematic review and meta-analysis. Clin Gastroenterol Hepatol.

[29] Tang LJ, Ma HL, Eslam M (2022). Among simple non-invasive scores, Pro-C3 and ADAPT best exclude advanced fibrosis in Asian patients with MAFLD. Metabolism.

[30] (2025). Liver biopsy. https://en.wikipedia.org/wiki/Liver_biopsy.

[31] Arab JP, Barrera F, Arrese M (2018). The evolving role of liver biopsy in non-alcoholic fatty liver disease. Ann Hepatol.

[32] Brunt EM, Kleiner DE, Carpenter DH (2021). NAFLD: reporting histologic findings in clinical practice. Hepatology.

